# Modulation of Gut Microbiome and Metabolome as One of the Potential Mechanisms of Ketogenic Diet Effect in the Treatment of Epilepsy

**DOI:** 10.3390/nu18010031

**Published:** 2025-12-21

**Authors:** Katarzyna Kowalcze, Damian Dyńka, Wiktoria Klus, Magdalena Dudzińska, Agnieszka Paziewska

**Affiliations:** 1 Institute of Health Sciences, Faculty of Medical and Health Sciences, University of Siedlce, 08-110 Siedlce, Poland; katarzyna.kowalcze@uws.edu.pl (K.K.); damian.dynka@uws.edu.pl (D.D.); 2 Sirene Medical Clinic, 02-796 Warszawa, Poland; 3 Warsaw Genomics S.A., 02-089 Warsaw, Poland; wiktoria.klus@onet.com.pl; 4 Pediatric Neurology Department, Chorzow General Hospital, 41-500 Chorzów, Poland; mdudzinskapl@gmail.com

**Keywords:** epilepsy, neurological diseases, neurology, microbiome, metabolome, microbiota, modulation, ketogenic diet, metabolites, intestines, intestinal bacteria, treatment

## Abstract

Background/Objectives: The over 100-year-old practice of using ketogenic diet (KD) in the treatment of epilepsy has consolidated its position as an effective therapeutic tool. The available publications suggest a significant influence of KD on gut microbiome and metabolome and, on the other hand, a correlation between microbiome and metabolome changes and the course of epilepsy. The conclusion is therefore justified that KD can exert a therapeutic effect in epilepsy through the mechanism of gut microbiome and metabolome modulation. Methods:This article is a narrative review aimed at a comprehensive analysis of the literature to gather existing evidence on the relationship between ketogenic diet, its antiepileptic effects and modulation of gut microbiome and metabolome. Results: It has been demonstrated that a ketogenic diet exerts a significant effect on intestinal bacteria and their metabolites, among other actions, increasing the *Bacteroides* to *Firmicutes* (B/F) ratio, alleviating dysbiosis, reducing the inflammatory condition in the gut and whole body, increasing the number of specific strains associated with antiepileptic effect, mediating the production of neurotransmitters (GABA, serotonin), exerting influence on the dopaminergic system, on a number of metabolic pathways, on inhibition of genotoxicity and production of short-chain fatty acids (SCFA) in the intestine. Conclusions: Further studies are needed, since the effect of KD on gut microbiome and metabolome modulation in the treatment of epilepsy is an extremely promising and trendsetting direction of research.

## 1. Introduction

The widespread occurrence of epilepsy significantly influences the quality of life of millions of people (mainly children) worldwide. That prompts researchers to conduct increasing numbers of studies and to publish papers on the treatment, pathophysiology, classification, or epidemiology of that disease [1,2]. One of the leading research areas in this respect is the therapeutic, antiepileptic effect of the ketogenic diet. It has been used with high effectiveness in the treatment of epilepsy for over 100 years, since an article by Wilder was published in the year 1921 [3]. Despite the high effectiveness of that treatment method, its mechanisms of action still have not been unequivocally confirmed. One of the possible mechanisms of ketogenic diet effect in the treatment of epilepsy is modulation of the gut microbiome and metabolome [4,5]. That potentially therapeutic mechanism is a new, highly prospective, and, possibly, trendsetting direction of studies on the treatment of epilepsy. The research papers published to date suggest a crucial role of microbiome, metabolome, integrity of the intestinal barrier and gut–brain axis, not only with respect to neurological diseases, but also in the functioning of the whole body [6,7,8,9,10,11].

The ketogenic diet is a nutritional model, the aim of which is the induction of the production of ketone bodies (i.e., β-hydroxybutyrate, acetoacetate, and acetone) in the human organism, leading to the state of ketosis. The state of ketosis in some measure mimics a period of fasting, since in both cases an increased production of ketones (which are produced from fats) takes place [6,12,13]. A ketogenic diet assumes supplying most energy from fat, limiting carbohydrate consumption to a maximum of 50 g daily, and a medium (or low) consumption of protein. The composition of macronutrients would be different for healthy individuals striving to achieve the state of nutritional ketosis compared to the ketogenic diet variant used in the treatment of epilepsy, the aim of which is to achieve a clinical state of ketosis. The strictly clinical variant of that diet contains a higher amount of fat (even up to 90% of daily energy supply), a lower amount of carbohydrates (10–20 g daily), while the remaining energy supply comes from protein (up to 10% share in daily energy supply). Such a composition allows for reaching a higher concentration of ketone bodies in blood and proves more effective in the treatment of epilepsy than the standard version of the ketogenic diet for healthy individuals [14,15,16,17]. It is not surprising, however, that the strictly clinical ketogenic diet variant is simultaneously fairly restrictive, and its observation in children may be problematic. Increasingly frequently, however, less restrictive diets are used, e.g., Modified Atkins Diet (MAD), which, as it turns out, also demonstrates antiepileptic effects [18].

It is worth making it clear that there is no definition of the ketogenic diet describing its qualitative composition. Besides the composition of macronutrients, significant freedom is seen in the selection of nutritional products. That is extremely important, since the fact of whether the ketogenic diet will exert a favourable or unfavourable effect on the human organism may depend on its composition. The diet composition is all the more important for the gut microbiome and metabolome [19,20,21]. The fact that a diet is ketogenic does not exempt it from being composed of natural, minimally processed foods. Ketogenic diets can also be of low quality and highly processed. It is well established that ultra-processed foods—characterized by high levels of synthetic additives and emulsifiers, and low fiber content—promote gut dysbiosis, increase intestinal permeability, and disrupt the gut–brain axis. For instance, consumption of ultra-processed foods has been associated with reductions in *Akkermansia muciniphila* and *Faecalibacterium prausnitzii*, alongside increases in pro-inflammatory microorganisms, potentially exacerbating intestinal inflammation [22]. Fiber content within a ketogenic diet is an important factor, as diets can be formulated to be either low or high in fiber, leading to different effects on the gut microbiome. For example, inclusion of fiber-rich foods promotes the growth of short-chain fatty acid (SCFA)-producing bacteria such as *Faecalibacterium*, *Eubacterium*, *Roseburia*, and *Blautia*, while modulating plasma and fecal metabolites, including increased propionic acid levels. As highlighted by Meiners et al. (2025), even simple qualitative changes in diet composition can have a substantial impact on the microbiome and metabolome [23]. The profile of dietary fatty acids also plays a significant role. Omega-3 fatty acids exert beneficial effects on the gut microbiome, whereas an excess of omega-6 relative to omega-3 appears to diminish the positive impact of omega-3 supplementation on microbial diversity and abundance, potentially promoting dysbiosis [24]. Even the presence and quantity of olive oil can influence microbial composition. Garrido-Romero et al. (2025) reported that bioactive compounds in olive oil enhance beneficial bacterial populations, including *Lactobacillus* and *Bifidobacterium*, suppress potentially pathogenic taxa, and promote SCFA production and other microbial metabolites, thereby strengthening intestinal barrier integrity [25]. Finally, the type of dietary protein also differentially affects the microbiome [26]. A recent meta-analysis confirmed that plant-based proteins may increase the abundance of *Bacteroides, Muribaculaceae*, and *Allobaculum*, whereas animal-based proteins are associated with higher levels of *Colidextribacter*, *Clostridium* sensu stricto 1, and *Rikenellaceae*_g [27]. All of this suggests that the qualitative composition of the diet itself can be a key determinant of its beneficial or detrimental effects on the gut microbiome and metabolome and therefore warrants emphasis.

## 2. Ketogenic Diet in the Treatment of Epilepsy

### 2.1. Importance of Ketogenic Diet in the Treatment of Epilepsy

Epilepsy is an extremely common neurological disease, affecting about 50 million patients worldwide. It manifests itself as recurrent attacks of convulsions caused by excessive electric discharges in neurons. The frequency of convulsive seizures in various individuals may vary, and they can occur both many times daily as well as sporadically (e.g., once a year). The first mentions of that disease reach back even to 4000 BC [28]. For as long as the diagnoses of epilepsy were being made, various therapies were used throughout the centuries. Initially (reaching back to BC times) and until the last century, observation of fasts was a frequently used method of treatment. That resulted from the fact that during the period of fasting, ketone bodies are produced, and the organism is brought into a state of ketosis. It has been observed that the mentioned state very effectively reduces the frequency of seizure attacks [29,30,31]. Unfortunately, the high frequency of fasts was leading to starvation and was inducing undesirable effects. In spite of favourable therapeutic effects in the form of reduction in epileptic seizure frequency, the patients were, however, asthenic. Therefore, that method could not have been used in long-term treatment. Only in the last century the use of ketogenic diet was started, since it was noted that similar therapeutic effects were being achieved, but without the negative consequences of starvation. The ketogenic diet, even today, is one of the main methods of treatment of epilepsy, since it shows such high effectiveness that in many cases is better than pharmacotherapy. It is helpful in states of drug-resistant epilepsy (DRE), and frequently it is the last possibility of therapy for children affected by the disease [3,6,32,33,34].

### 2.2. Possible Therapeutic Mechanisms of Ketogenic Diet in Epilepsy

The mechanisms of the therapeutic effects of ketogenic diet still remain unclear. Influences have been postulated, among other effects, through reduction in glucose concentration, increase in ketone concentration, providing alternative energy supply for the neurons (through monocarboxylate transporters (MCTs) present in the nerve cells), neuroprotective effect, improvement of mitochondrial functioning, effect on mitochondrial structures, anti-inflammatory and antioxidant effects, reduction in demyelination and of death of oligodendrocytes, intensification of macroautophagy in the brain, prevention of gathering of autophagosomes, reduction in beta-amyloid amount, reduction in microglia activation, effect on the blood–brain barrier (BBB), effect on the neurotransmitters, increased glutamate conversion to glutamine, increase in gamma-aminobutyric acid (GABA) concentration, effect on the activity of ATP-sensitive potassium (KATP) channels, effect on the concentrations of biogenic amines (including dopamine, serotonin, noradrenaline) [35,36,37,38,39,40,41].

In spite of a number of potential mechanisms of action, it has not been unequivocally confirmed that any of them can directly exert an anticonvulsant effect. The most thought-provoking is, however, the fact that the anticonvulsant effect is not a result of a mere increase in ketone bodies. It has already been shown many years ago that the anticonvulsant effect is not always correlated with the amount of ketone bodies in blood serum, urine and exhaled air. In view of that, an increase in ketone concentration is not proportional to a reduction in the frequency of seizure episodes [40,42,43,44,45,46,47].

Taking the above into consideration, the answer to the question is still sought: how a ketogenic diet exerts an antiepileptic effect in the treatment of epilepsy. A very promising mechanism is the effect through gut microbiome and metabolome modulation. It is explained in the chapters below.

## 3. Gut Microbiome and Metabolome

### 3.1. Gut Microbiome

Microbiome is the collection of all microorganisms (including bacteria, fungi, viruses and their genes) colonizing the body. It is related to their biological functions in a given environment [48,49,50]. Gut microbiome is therefore the collection of microorganisms in the intestines, where the greatest number in the whole body is present (mainly in the large bowel). It is estimated that the number of microorganisms in the human gut reaches 100 trillion, including over 1000 bacterial species [51,52,53,54,55,56]. The main phyla of bacteria colonizing the colon include Gram-positive *Firmicutes* (mainly *Ruminococcaceae* and *Lachnospiraceae*, and less numerous *Lactobacillus* genus) and Gram-negative *Bacteroidetes*. These two bacterial phyla account for 90% of the intestinal microbiota. The bacterial phyla most frequently colonizing the large bowel also include *Actinobacteria* (e.g., from the *Bifidobacterium* genus), *Proteobacteria* and *Verrucomicrobia* (mainly from the *Akkermansia* genus) [53,57,58,59].

Gut microbiome is one of the key factors influencing the health condition, and it performs a number of functions, including digestion of nutrients, supporting the immune system, production of some vitamins (e.g., K1 and some B-group vitamins), production of metabolites (e.g., short-chain fatty acids (SCFA)), maintaining intestinal integrity, regulation of inflammatory conditions, participation in the production of neurotransmitters and bidirectional communication with the brain through the gut–brain axis, so it also influences mental health [60,61,62,63,64,65,66,67,68]. It also exerts a significant effect on the nervous system and is related to the course of epilepsy [69,70]. It is equally known that it can be modulated by diet [71,72].

### 3.2. Gut Bacterial Metabolome

Gut bacterial metabolome is a collection of all metabolites produced by the gut microbiome. They include, among other compounds, SCFAs, in which the main role is played by butyric acid (BA), acetic acid (AA) and propionic acid (PA). Other SCFAs include isobutyric acid (IBA), valeric acid (VA), caproic acid (CA), isovaleric acid (IVA). They constitute a type of communicator between the microbiome and the immune system, regulate inflammatory conditions, serve as a fuel for the intestinal epithelium, and enhance the intestinal barrier function, and should be regarded as an integral part of the gut microbiota [73,74,75]. The important metabolites also include trimethylamine (TMA) derived, among other compounds, from choline, betaine, glycerophosphorylcholine (GPC) or carnitine, and trimethylamine N-oxide (TMAO) derived from TMA [53,76,77,78]. Bacteria also produce amino acids, including glutamic acid, which is a substrate for the production of GABA, i.e., an important neurotransmitter [79]. Moreover, intestinal bacteria can metabolize tryptophan, among other metabolites, to scatole or serotonin [80,81], although over 90% of serotonin is produced in the intestinal cells [82].

## 4. Interactions of the Microbiome and Metabolome with the Course of Epilepsy

### 4.1. Association of Dysbiosis and Other Gastrointestinal Disorders with the Risk of Seizure Development

A number of studies have confirmed the interactions of gut microbiome, and thus metabolome, with epilepsy [83]. The relationship is so significant that it has been postulated that microbiome modulation can exert preventive and therapeutic effects on epilepsy, what has been increasingly frequently discussed in scientific publications [69,71,84,85,86]. That is not surprising, however, taking into account the results of studies suggesting an increased risk of seizures resulting from the use of antibiotics [87,88]. The potential mechanism is related to the influence of the microbiome of the gut–brain axis and modulation of neurotransmission, that includes antagonism and inhibition of GABA synthesis and agonistic effect on the N-methyl-D-asparagine receptor. They simultaneously decrease the seizure threshold [84,89,90,91]. On the other hand, studies are available suggesting a reduction in the number of seizure episodes as a result of the administration of antibiotics. The authors present six cases of patients with drug-resistant epilepsy who became temporarily seizure-free during treatment with antibiotics. Two weeks after discontinuation of the antibiotics, the anticonvulsant effect disappeared [92]. In view of that, the data on whether antibiotic therapy enhances seizures or prevents them still varies. Most likely, a short-lasting change in the microbiome through administration of antibiotics can temporarily reduce the frequency of seizure episodes, but in the long run, it can even potentiate them, what has been shown in the mentioned studies. In either case, we can conclude, however, that intestinal microbiota plays an extremely important role in epilepsy; if it changes because of antibiotic therapy it changes the course of the disease. That has also been confirmed by the effect of the administration of probiotics. Gómez-Eguílaz et al. tried to study the efficacy of the administration of probiotics in controlling epileptic seizures in patients with DRE. After four-month-long supplementation with probiotics, with a combination of *Lactobacillus, Bacteroides* and *Streptococcus* subspecies, it was demonstrated that in 28.9% of patients a reduction occurred in the number of seizure episodes by over 50% and a significant improvement in quality of life was reported [93]. El-Sharkawy et al. published studies with a similar end result in patients with DRE. Almost 43% of patients achieved over 50% reduction in the number and duration of seizures after probiotic compared to pre-probiotic data [94].

The relationship between gut dysbiosis and seizure attacks has also been confirmed in the study by Huang et al. It demonstrated that the development of mild infantile seizures was preceded by mild gastroenteritis [95]. Dysbiosis can therefore be regarded as an inherent element in the course of epilepsy [96,97,98]. Seizure attacks are also associated with gastroesophageal reflux disease (GERD) [69,99,100], peptic ulcers (which, as demonstrated, occur eight times more frequently in epileptic patients than in the general population) [101] or celiac disease (in 10% of patients with celiac disease, neurological complications develop, including convulsions, while in some patients with epilepsy, celiac disease develops (in 0.78–9.1%)) [69,102,103]. Moreover, irritable bowel syndrome (IBS) increases the risk of epilepsy development, while epilepsy itself increases, even five times, the risk of IBS (compared with the control group) [98,104,105]. It has also been demonstrated that epileptic seizures can be observed in the course of unspecific inflammatory bowel diseases (IBDs) [69,106] and even in gastrointestinal tract infections, in which such epileptic activity is called “situation-related seizures” [107]. That shows the great number of various gastrointestinal disorders/diseases, which are associated with increased risk of seizures [69,108].

### 4.2. Characteristics of Microbiome and Metabolome in Epileptic Patients

It has been found that epileptic patients show characteristically changed gut microbiota. In an increasing number of studies an increase in the *Firmicutes, Proteobacteria, Verrucomicrobia* and *Fusobacteria* phyla is demonstrated in epileptic patients, while in the *Bacteroidetes* and *Actinobacteria* phyla a decrease occurred [109,110,111]. Importantly, in patients with DRE a particularly changed microbiome is present, compared not only with healthy individuals, but also with patients with drug-sensitive epilepsy (DSE) [112,113]. The available studies on microbiome characteristics in epilepsy show a difference between the group of healthy individuals, the group with DSE and patients with DRE. Gong et al. tried to study the difference in gut microbiota between healthy individuals and epileptic patients, by means of 16S ribosomal RNA sequencing. It was found that the diversity of alfa microbiota in epileptic patients was lower compared to the healthy control group. The mean number of species found in epileptic patients was 275.33 ± 41.64, while in the healthy controls that number was 347.26 ± 102.40. It is worth mentioning, however, that in both groups the diversity profile was typical of healthy individuals, i.e., dominated by *Firmicutes, Bacteroidetes, Actinobacteria, Proteobacteria* phyla [114]. However, in patients with epilepsy, an increase in *Actinobacteria* and *Verrucomicrobia* and a decrease in *Proteobacteria* were observed. On the other hand, in the same patients at genus level, decreases in *Klebsiella, Sutterella, Escherichia-Shigella, Lachnospiraceae-NK4A136*-group and *Lachnoclostridium,* and increases in *Prevotella-9, Blautia, Bifidobacterium, Akkermansia, Megamonas, Ruminococcaceae UCG-014, Ruminococcus gnavus group, Romboutsia* and *Eubacterium halii* group were observed. Importantly, in patients with DRE (compared to those with DSE and the healthy control group) an increase in *Actinobacteria, Verrucomicrobia, Nitrospirae* and *Firmicutes* and a decrease in *Cyanobacteria* were found. At the genus level, an increase in *Blautia, Bifidobacterium, Subdoligranulum, Dialister* and *Anaerostipes* and a decrease in *Parabacteroides* occurred in patients with DRE. Yuwattana et al. conducted a similar study in which were demonstrated characteristic changes in gut microbiota in children with epilepsy that correlate with response to treatment. In the case of DSE patients, *Saccharimonadales* and *Peptocostridium* increased significantly, compared to the DRE, in which is a noticeable abundance in *Vibrionaceae*, especially *Enterobacter, Grimontia* and *Rhodobacteraceae* [115]. In a different study, Riva et al. presented that no significant phylum-level differences were found in epilepsy patients and control groups, which consisted of healthy cases. The difference, though, was found in the genus-level related not only to epilepsy and control groups but also between DRE and DSE. In this case, we can suspect that subtle changes in bacterial populations are associated with resistance to medication. In DRE, there was a noticeably higher abundance of Hung, which is anaerobic bacteria which belong to *Clostridiaceae* family and is highly resistant to antibiotics. Decreased in DRE, compared to DSE, was *Eubacterium* spp., which are SCFA producers; some of them produce GABA. Still longitudinal studies are necessary to enable the distinction between predictive biomarkers and treatment-induced changes [116]. These studies suggest that differences in gut microbiome can be used as a DRE-predictive biomarker. On the other hand, another study [117] demonstrated an increased diversity alpha in patients with DRE, particularly in the group of patients with four or more seizures yearly (compared to those with fewer than four seizure episodes yearly). In the group of patients with four or fewer seizures yearly, an increase in Bifidobacteria and Lactobacillus was found. In DRE patients, an increase was observed in bacteria belonging mainly to the *Firmicutes* phylum, i.e., *Roseburia, Coprococcus, Ruminococcus* and *Coprobacillus*, compared to the DSE group. Interestingly, the composition of gut microbiota in patients with DSE was similar to that in healthy control individuals. In another study [118], the alpha diversity in children with epilepsy was lower compared with the healthy control group (parents). In the paediatric patients, an evidently lower total number of observed metagenomic operational taxonomic units (mOTU), lower total species richness Chao1 and Shannon evenness index were noted. Furthermore, the differences between the samples from epileptic children were, as a rule, higher compared with the samples from the control group (measured as beta diversity). In another study, the authors demonstrated significant differences in gut microbiota between infants with DRE and healthy infants. Based on Shannon analysis, a higher diversity of microbiota was found in healthy infants than in those with DRE. The principal component analysis (PCA) of gut microbiota profile demonstrated also that epileptic infants can be clearly distinguished from the healthy control group [119]. Comparing the quantitative composition of microbiota in babies with DRE, the greatest share was of the *Firmicutes* phylum (45.82%), followed by *Bacteroidetes* (26.75%), *Proteobacteria* (24.34%), *Actinobacteria* (2.38%), *Verrucomicrobia* (0.59%) and *Fusobacteria* (0.43%). On the other hand, at genus level, in the group of patients, *Cronobacter* predominated (23.30%, compared to 0% in the control group), while in the healthy control group *Bacteroides* organisms were most prevalent (42.68%) (compared to 17.93% in the DRE group). In the healthy group, higher percentages were also observed of *Prevotella* (7.29% vs. 0.37%) and *Bifidobacterium* (7.84% vs. 0.91%). Cui et al. compared the fecal microbiome composition in epileptic patients with that in the healthy control group. The differences were significant [120]. The alpha diversity was higher in healthy individuals, while the Venn diagram showed that, on the other hand, in epileptic patients, more unique operational taxonomic units (OTUs) were present. At phylum level, in the group of patients *Proteobacteria* and *Actinobacteria* increased significantly, while in the healthy group, an increased relative abundance of *Bacteroidota* was observed. In healthy individuals, compared to patients, 59 genera were increased, including *Bacteroides, Megamonas, Prevotella, Lachnospiraceae* (non-classified) and *Blautia*, while on the other hand, the microbiota of patients was enriched by 23 genera (*Faecalibacterium, Escherichia-Shigella, Subdoligranulum and Enterobacteriaceae* (non-classified)). In another study [121], no significant differences in alpha and beta diversity were demonstrated between both groups of adult epileptic patients (DRE and DSE). It was, however, demonstrated that in the DSE group, the relative abundance of *Bacteroides finegoldii* and *Ruminococcus*-g2 increased, while in the DRE group, an increased relative abundance of *Negativicutes* was shown. In a 2023 study, the microbiome of children with non-epileptic cerebral palsy (NECP) was compared with that in children with cerebral palsy and epilepsy (CPE) [122]. Epileptic patients were characterised by significantly lower abundance of *Bacteroides fragilis* and *Dialister invisus*, and higher abundance of *Phascolarcobacterium faecium* and *Eubacterium limosum*. In the CPE group, the patients with DRE were characterised by significantly higher abundance of *Veilonella parvula*. Another 2023 study clearly demonstrated that the microbiome of epileptic patients compared to that of healthy individuals differed at the levels of genus, family, order, class and phylum. *Flavihumibacter, Niabella, Anoxybacillus, Brevundimonas, Devosia* and *Delftia* were only present in healthy individuals. On the other hand, *Megamonas* and *Coriobacterium* were only found in the group with epilepsy. The authors, at the same time, indicated these two bacterial genera from *Actinobacteria phylum* as biomarkers useful in the diagnosis and follow-up of epilepsy [123]. Interesting results were obtained by the authors of a publication from 2022, who studied the correlation between gut and oral microbiota in children with CPE. It was revealed that CPE children had reduced levels of *Firmicutes* and *Bacteroides* and increased levels of *Actinomycetes* in the oral cavity (compared with healthy children). The three most numerous bacterial genera in the oral cavity of CPE children were *Prevotella, Fusobacterium* and *Neisseria*, while those in the gut microbiota included *Bifidobacterium, Bacteroides* and *Prevotella*. The authors plainly said that oral and gut microbiota were strictly related to each other. The dysbiotic oral microbiota itself can migrate to further segments of the gastrointestinal tract and contribute to intestinal dysbiosis [124].

A change in the microbiome frequently brings about a change in the bacterial metabolome and, thus, in the metabolites, which are produced by the microbiota, and in other metabolites present in the intestine. In a publication from 2023, the authors compared not only the gut microbiome but also metabolome between patients with and without epilepsy, while both groups were also affected with cerebral palsy. In the group of epileptic patients, increased concentrations were found of metabolites, i.e., kynurenic acid, L-vinic acid, D-saccharic acid, 2-oxinol, dopamine, 2-hydroxyphenylalanine and 3,4-dihydroxyphenyloglycol. The patients struggling with DRE, on the other hand, were characterised by increased levels of indole and homovanillic acid. The epileptic patients also had an increased number of ethanol production pathways, but reduced numbers of pathways of serine, glutamate, quinolinic acid, glycerol degradation and of sulphate and nitrate reduction. The authors called attention to the important role of the gut–brain axis in the results obtained [122,125]. Besides that, a negative correlation was demonstrated of *Bacteroides fragilis* with kynurenic acid concentration, similarly as *Phascolarctobacterium faecium* was inversely correlated with dehydroascorbic acid concentration, while *Dialister invisus* was positively correlated with the pathway transforming acetyl-CoA into acetate. A publication from 2022 also points to the role of gut microbiota metabolites in the form of short-chain fatty acids (SCFA) in the course of epilepsy, dividing their effect on the proneness to seizure into three mechanisms. The authors stress their effect on the modulation of excitatory and inhibitory neurotransmitters, on nervous system inflammation, oxidative stress and psychosocial stress [69,126]. TMAO metabolites can also play a role in neurological diseases, although that domain is still poorly studied [127,128].

The characteristics of the microbiome and metabolome in epileptic patients are presented in Table 1.

## 5. Modulation of Gut Microbiome and Metabolome as a Potential Therapeutic Mechanism of Ketogenic Diet in Epilepsy

The many-year-long practice of ketogenic diet use in the treatment of epilepsy has well established its position as an effective therapeutic tool. However, the potential mechanisms in which the ketogenic diet shows its therapeutic effect are still being investigated [36,129,130]. Among them, the influence on the modulation of gut microbiome and metabolome emerges as one of the main mechanisms of ketogenic diet effect in epilepsy [131,132,133].

### 5.1. Effect of Ketogenic Diet on the Modulation of Gut Microbiome and Metabolome in Epileptic Patients

An increasing number of publications suggest a significant effect of ketogenic diet on the modulation of gut microbiome and metabolome in the treatment of epilepsy. The last few years have provided abundant scientific evidence linking the gut–brain axis to epilepsy. Both animal and human studies suggest that the antiepileptic effects of KD can be controlled by microbiota-dependent devices. It is hypothesized that gut microbiota modification may be an effective alternative to the development and severity of epilepsy. This is particularly true for the abundance of certain bacteria, such as *A. muciniphila* and *Parabacteroides gordonii* [87,134]. Several studies on human epilepsy, but also in animal models, have shown that KD alters the gut microbiome [135,136,137]. The gut microbiome has been shown to modulate the anticonvulsant effects of the ketogenic diet, but the detailed mechanism is still poorly understood [4]. In recent years, a relationship between the gut microbiome and the KD has been identified on various host factors, including metabolism and lipid metabolism [138], cell function [139,140], brain function [87], and behavior [141]. It has been demonstrated that KD alters the gut microbiome in several human epilepsy studies, but also in animal models [135,137]. In a study of 2023, the authors analyzed fecal samples from patients with mitochondrial epilepsy before and after three months of ketogenic diet (KD). It has been proven that KD can affect the composition, diversity and functions of gut microbiota in epileptic patients [142].

As a result of KD application, a reduced abundance of *Firmicutes*, and an increased abundance of *Bacteroidota* were observed. An increased abundance of *Bacteroides* was also noted (particularly *Bacteroides fragilis*), while in the control group (CD) *Actinobacteriota* and *Phascolarctobacterium* significantly predominated. The *Firmicutes* phylum predominated in both groups, but its share in the KD group was lower (42.76% in KD vs. 48.13% in the CD group). The share of the *Bacteroidota* phylum increased in the KD group to 36.93% compared with 25.41% in the CD group. On the other hand, in the KD group, lower percent values were noted of *Actinobacteriota* (1.66% vs. 7.64% in CD), *Fusobacteriota* (0.68% vs. 1.65% in CD) and *Desulfobacterota* (0.15% vs. 0.50% in CD). At the genus level, *Bacteroides* (mainly through a significant increase in the count of *Bacteroides fragilis* species) increased significantly in the KD group (28.78%) compared to CD (9.51%). In the KD group an increase was observed in *Blautia.s_Blautia_sp*_N6H1 at the genus level and in *Anaerotignum_lactatifermentans* at the species level. Differences were demonstrated at three levels of the Kyoto Encyclopedia of Genes and Genomes (KEGG). Although the differences were observed at each level, they particularly concerned 12 important pathways at level 3 and some pathways at level 2. In the KD group, an increased enrichment was noted in pathways, i.e., citrate pathway (TCA), pertussis pathway, biosynthesis of penicillin and cephalosporins, biosynthesis of lysosomes and glycosphingolipids, phosphatidylinositol signalling system, biofilm formation (*Escherichia coli*). At the same time, a reduced enrichment in the KD group was observed in pathways including Quorum sensing, Legionellosis, nicotinate and nicotinamide metabolism, arginine biosynthesis, or bacterial secretion system. It was also noted that phenylalanine metabolism dropped, while biosynthesis of phenylalanine, tyrosine and tryptophan showed an increase after ketogenic diet application [142]. It should also be noted that some data from recent studies are inconsistent. In one study, Lee et al. analyzed stool samples from a population of eight Korean children aged 1–7 years with intractable epilepsy and 32 age-matched healthy controls. Using a 16S rRNA gene sequencing approach, they found that α-diversity was higher in epilepsy patients, while β-diversity revealed a clear difference in bacterial composition between these groups. In the epilepsy group, the number of *Bacteroidetes* bacteria was lower, and the number of *Actinobacteria* was higher than in the healthy group. Species biomarkers for intractable epilepsy included the *Enterococcus faecium* group, the *Bifidobacterium longum* group, and *Eggerthella lenta*. Analysis of these data confirmed that patients with intractable epilepsy had gut bacterial dysbiosis [143]. In a subsequent study, Lee et al. conducted an exploratory study, but this time in adult patients. They prospectively enrolled 44 adult patients with epilepsy and classified them into drug-responsive and drug-resistant groups but found no differences in α or β diversity between these groups. While the abundance of *Firmicutes, Bifidobacterium, Shigella, Veillonellales, Klebsiella*, and *Streptococcus* increased in patients with drug-resistant epilepsy (DRE), the relative abundance of *Bacteroides, Ruminococcus_g2*, and *Bifidobacterium* was increased in patients with DSE [121]. The significant difference in gut microbiota composition between patients with DRE and DSE confirmed the findings of other studies by Peng and Gong. These studies suggest the presence of microbial dysbiosis in patients with DRE, indicating the potential value of using gut microbiota as a sensitive biomarker for diagnosis and treatment target to improve seizure control [117]. It is important to emphasize that most studies indicated that α-diversity in the HC group was higher than in the DRE group. However, Peng’s study [117] showed the opposite trend, with increased α-diversity in the DRE group, indicating that microbiota changes in epilepsy patients may not always be consistent. Therefore, further analysis is needed in a larger group size, considering age, diet, living environment, and other factors influencing the gut microbiota.

The effect of ketogenic diet on, among other things, microbiome and gut inflammatory conditions constituted a part of another study from 2023, conducted in epileptic children. It was found that, despite a lower diversity of microbiota in children with DRE treated with ketogenic diet, the level of S100A12 (a marker of enteritis) was within the normal range. That showed that a reduction in bacterial diversity alone did not affect intestinal inflammatory condition, even for a longer period of time (the median duration of ketogenic diet application = 15 months) [144]. In 2023, an interesting study was also conducted in beagle dogs, in two groups: with idiopathic epilepsy (IE) and healthy. Both groups were fed ketogenic diet enriched with medium-chain triglycerides (MCT) for one month. The results differed from those observed in humans, since epileptic dogs had significantly higher numbers of species observed both before and after ketogenic diet application, compared with healthy dogs (also confirmed by Chao1 index—significant differences). Interestingly, the initial microbiota composition in dogs with DSE was similar to that in healthy animals. In these groups, the diet increased the abundance of *Bacteroidetes* and *Fusobacteria* and reduced the abundance of *Firmicutes*, contrary to the situation in the case of DRE. The authors demonstrated that after MCT administration with the diet, a tendency was observed towards a reduction in the differences in the microbiota between dogs with DSE and those with DRE [145]. Another, earlier published study revealed that observation of ketogenic diet enriched with MCT caused an increase in alpha diversity, an increase in the abundance of an unnamed *Bacteroidaceae* species of the 5-7N15 genus, an increase in the abundance of unclassified members of the *Erysipelotrichaceae* and *Fusobacteriaceae* families, a decrease in *Blautia* sp. and *Megamonas* sp, and no changes in beta diversity [146]. The authors of a 2020 study investigated the relationship between gut microbiota and systemic inflammatory conditions in children with DRE. It was demonstrated that bacteria, i.e., *Gordonibacter pamelaeae, Eggerthella lenta, Lactococcus lactis* and *Bifidobacterium longum* susp. Longum, were associated with an anticonvulsant response to ketogenic diet, while *Alistipes shahii* and *Eubacterium rectale*, among other organisms, were not related to such a response. Importantly, the values of 26 inflammatory condition markers decreased in children on a ketogenic diet (among other markers: IL-17A, IL-17C, TNF, IL-12B, IL-18R1 and GDNF), and only three parameters increased (CCL25, IL-18 and IL-1 alpha). It was revealed that *B. longum* and *B. breve* were related to TNF-alpha. All three parameters were increased in children, who later responded with a reduced frequency of seizures (compared to children not responding to KD). Significant negative correlations with CCL25 were shown by *B. kashiwanohense PV20-2, B. angulatum GT102, B.* adolescentis ATCC 15703 and *B. breve JCM 7019* [147]. In 2021, the first study (according to the authors) was conducted, assessing the effect of a one-month-long ketogenic diet on the intestinal environment in epileptic patients through an analysis of short-chain fatty acid (SCFA) production and fecal water toxicity. It was demonstrated that KD significantly reduced the total amount of SCFA (from 20.7 mg/g on average, to 9.3 mg/g), acetate (from 8.4 mg/g on average, to 2.7 mg/g) butyrate (from 4.8 mg/g on average, to 3.2 mg/g), propionate (from 3.4 mg/g on average, to 2.4 mg/g), isovalerate (from 1.1 mg/g on average, to 0.6 mg/g) and isobutyrate (from 0.6 mg/g, on average, to 0.3 mg/g). At the same time, the median fecal water genotoxicity significantly decreased after KD application, contrary to the cytotoxicity level, which showed no significant change after the intervention. Importantly, in all patients simultaneously, an improvement occurred in the form of >50% reduction in seizures [148]. Several months later, another study was published [149], in which another group of researchers tried to study the correlation between gut microbiome an SCFA in the feces of children with DRE. Importantly, after six months on a ketogenic diet, the SCFA content in feces increased, and that result was different from that in the previous study lasting one month [148]. After six months on a ketogenic diet the abundance of *Subdoligranulum, Dialister, Alloprevotella* increased, while that of *Bifidobacterium, Akkermasia, Enterococcaceae* and *Actinomyces* decreased. Importantly, too, a higher alpha diversity in the DRE group and a significant increase in the *Actinobacteria* phylum and the *Enterococcus, Anaerostipes, Bifidobacterium, Bacteroides* and *Blautia* genera were demonstrated. Among other effects, a positive correlation was revealed of *Parabacteroides* and *Lachnoclostridium* with acetic acid, and of naerostipes and Chujaibacter with butyrate, which, in turn, was negatively correlated with *Delftia. Fusicatenibacter* showed a strong correlation with propionic acid while *Collinsella* was correlated with isobutyric acid and (together with *Olsenella*) positively correlated with isovaleric acid. The authors clearly concluded that ketogenic diet could alleviate epilepsy-related dysbiosis [149]. It is also not clear how KD can affect TMAO metabolites in epilepsy, since, possibly, such activity could also exert an effect on the course of that disease [128]. Lindefeldt et al. assessed gut microbiota changes in children with DRE-fed ketogenic diet for three months, compared with the control group (their parents). It was demonstrated that alpha diversity in the DRE group was reduced already before the diet began (compared to the control group), and after ketogenic diet application, that difference further increased (mOTU, Chao1 and Shannon indices). Ketogenic diet led to a reduction in the relative abundance of Actinobacteria (mainly Bifidobacterium, particularly two species: *Bifidobacterium longum* (reduction from 8.1% to 2.4%) and *B. adolescentis* (reduction from 3.2% to 0.2%)) and an increase in Proteobacteria. Moreover, after three months on KD a reduction in the abundance of *Eubacterium rectale* (from 2.5% to 0.5%) and *Dialister* genus (from 2.2% to 0.4%) and an increased abundance of *Escherichia* genus (from 3.1% to 8.5%) (mainly *Escherichia coli*) occurred. The decreased abundance of *Eubacterium rectale* was associated with a possible reduction in butyrate production (since it is the main butyrate-producing species), but ketogenic diet demonstrated no significant effect on the relative abundance of the butyrate production pathways (acetyl-CoA, glutarate, 4-aminobutyrate and lysine pathways). Besides that, the observation of KD led to changes in 29 SEED subsystems (among other changes, a reduction in seven pathways engaged in the metabolism of carbohydrates) [118]. Zhang et al. studied the characteristics and composition of gut microbiota in children with DRE before and after six months on ketogenic diet. KD caused a reduction in alpha diversity of gut microbiota, increased the abundance of *Bacteroidetes* and significantly decreased the abundance of *Firmicutes*. In the group not responding to KD, an increase occurred in the abundance of *Clostridiales, Rikenellaceae, Lachnospiraceae, Ruminococcaceae*, and *Alistipes* (compared to individuals responding to the treatment). That study showed that KD can demonstrate various effectiveness, depending on the change in gut microbiota composition and, thus, a specific microbiota may be a biomarker of treatment effectiveness in DRE [150]. Xie et al. studied how ketogenic diet changed the gut microbiota in children with DRE. After a week of KD application, at phylum level an increase was demonstrated in *Bacteroidetes* (38.71% vs. 26.75% before) and Fusobacteria levels (0.32% vs. 0.09% before) and a reduction in Proteobacteria (10.77% vs. 24.31% before). The *Firmicutes* level remained relatively stable (47% vs. 45.82% before). At the genus level, after a week on KD a significant decrease was observed of the percentage of *Cronobacter* (10.44% vs. 23.30% before), *Erysipelatoclostridium* (4.89% vs. 8.67%), *Faecalibacterium* (4.41% vs. 8.59% before) and other organisms, including Streptococcus, *Alistipes, Veillonella, Bifidobacterium, Lachnoclostridium, Lactobacillus*. On the other hand, the percentages increased of *Bacteroides* (24.42% vs. 17.93%), *Blautia* (7.69% vs. 2.57%), *Gemmiger* (5.05% vs. 1.92%), *Dysgonomonas* (5.36% vs. 1.49% before) and other bacteria, including *Anaerostipes, Prevotella, Dorea* and *Odoribacter* [119]. In a study by Tang et al. [133], the growth dynamics of gut bacteria were examined in a ketogenic diet. *Akkermansia muciniphila*, a bacterium strongly linked to the therapeutic benefits of the ketogenic diet, exhibited one of the highest growth rates, aligning with its markedly elevated abundance. The authors of this study suggest that analyzing bacterial abundance and growth dynamics could help deepen our understanding of the ketogenic diet-induced changes in gut microbiota and the therapeutic effects of this special diet [133].

The effect of ketogenic diet on the modulation of gut microbiome and metabolome in epileptic patients is shown in Table 2.

### 5.2. Potential Mechanisms of the Antiepileptic Effect of Ketogenic Diet Exerted Through Microbiome and Metabolome

Ketogenic diet, modulating the gut microbiome and metabolome, potentially contributes to antiepileptic effect via a number of mechanisms. The first of them is an alleviation of the dysbiosis itself, which is correlated with the course of epilepsy. Ketogenic diet alleviates dysbiosis, and thus the more visible its effect is, the more intense is the initial dysbiosis, as has been demonstrated in patients with DRE (the microbiome of whom significantly differs not only from that in healthy individuals, but even in patients with DSE). A correlation has been found between microbiome change due to KD application and the frequency of seizures. After KD discontinuation, the antiepileptic benefits rapidly disappear and are correlated with microbiological changes in the intestine ([96,111,113,117,143]. It has been shown that ketogenic diet modulates the microbiome mainly through increasing the *Bacteroidetes* to *Firmicutes* (B/F) ratio and, in some cases, reducing *Proteobacteria* [151]. In one study, a correlation was directly demonstrated between bacteria, i.e., *Gordonibacter pamelaeae, Eggerthella lenta, Lactococcus lactis, Bifidobacterium longum susp. longum,* and antiepileptic response to KD, while lack of response was associated, among other organisms, with *Alistipes shahii* and *Eubacterium rectale* [147]. Therefore, a direct correlation is present between the efficacy of KD and the effect on the gut microbiome.

Another mechanism includes KD effect on the production of neurotransmitters with the participation of intestinal bacteria. Gamma-aminobutyric acid (GABA) is the most important inhibitory neurotransmitter in the brain, and it plays an important role in the development and progression of epilepsy [152]. As it has been revealed, it can be produced not only in the brain but also in the intestine. It has been demonstrated that a number of intestinal bacteria, but mainly *Bacteroides, Parabacteroides* and *Escherichia,* can produce GABA (in these species, the pathways of GABA production are actively expressed) [153]. Interestingly, these bacterial genera are significantly less abundant in epileptic patients than in healthy individuals, and in patients with DRE, they are even less abundant than in those with DSE [117,119,120,122]. It has been found that application of KD increases the levels, particularly of those intestinal bacteria, which produce GABA, and at the same time, a correlation is observed with a reduction in seizure attacks [87,118,119,143,154]. That has been strongly confirmed by the fact that in epileptic patients, among other findings, a reduced number of glutamate (an excitatory neurotransmitter) degradation pathways is observed, which indirectly reduces GABA synthesis [122]. KD, through its effect on GABA, can also potentiate the activities of certain antiepileptic drugs (i.e., benzodiazepines) [155]. A ketogenic diet can also, through the microbiome, exert an effect on the metabolism of tryptophan and, thus, on the synthesis of serotonin (an important neurotransmitter) and kynurenine, important for the course of epilepsy. Serotonin levels are related to epilepsy, since their increase is associated with a reduction in seizures [81,156,157,158,159], while higher frequency of seizures and more severe course of epilepsy are associated with a reduced concentration of that neurotransmitter [160]. It was demonstrated that KD in epileptic children led to a significant reduction in kynurenine (KYN) concentration (30–57%) compared with the initial values and, at the same time, to a significant increase in kynurenic acid (KYNA) concentration (38–96%), without any significant effect on 3-OH-kynurenine (3-OH-KYN) levels. Besides that, a reduced tryptophan concentration was found. Higher KYNA and lower KYN concentrations were determined in patients, in whom a greater reduction in seizure frequency was obtained on a ketogenic diet [161]. The impact of the KD on tryptophan metabolism and the kynurenine pathway clearly warrants further investigation. This stems in part from evidence that elevated levels of quinolinic acid (QA) reflect increased activity within the kynurenine pathway. QA (along with indoxyl sulfate) is a neurotoxic metabolite of tryptophan, and impaired QA degradation may contribute to reduced levels of nicotinamide adenine dinucleotide (NAD^+^). As a key redox coenzyme, NAD^+^ plays a central role in cellular energy production by supporting oxidative phosphorylation. Some researchers have suggested that inhibition of quinolinate phosphoribosyltransferase (QPRT) during inflammation or chronic stress may lead to QA accumulation. Consequently, QA can build up in various organs and tissues, including the central nervous system, where it may disrupt neurotransmission and precipitate seizure recurrence [34,162,163]. Although QA levels can increase after consumption of tryptophan-rich protein, the clinical relevance of this mechanism remains uncertain. Further studies are needed to clarify how these metabolites interact with the ketogenic diet and whether they influence its antiepileptic efficacy. It is also known how important a role is played by the neurotransmitter dopamine and the dopaminergic system, which are disturbed in the course of epilepsy [164,165,166]. In an animal model, the authors have demonstrated that KD changes the activity of the mesocortical dopaminergic pathway, which, as they directly suggest, may have an effect on the therapeutic action of the diet, including reduction in the activity of seizure episodes [167]. It has been found that dopamine levels can be affected directly by the microbiota [168]. The authors of another publication plainly demonstrated bacterial strains able to produce dopamine. They mentioned, among other bacteria, *Escherichia coli*, the abundance of which increases due to ketogenic diet effect, and which also influences noradrenaline production [118,169]. It is therefore possible that KD modulates the microbiome in epileptic patients in a way leading to an improvement of dopaminergic system functioning, which can indirectly impact the frequency of seizures. Hampton et al., in their publication, suggest the potential cellular and molecular mechanisms, owing to which the interactions between specific bacteria modulate the peripheral metabolites, which in turn exert influence on the level of hippocampal neurotransmitters [118,170]. As mentioned in another publication, intestinal bacteria modulate the excitability of neurons and frequency of seizures, mainly through releasing neurotransmitters and inflammatory condition mediators, so a modulation of the microbiome can produce benefits in epilepsy [171].

The anti-inflammatory effect of ketogenic diet on the gut environment is another mechanism that translates to a number of benefits (including anti-inflammatory ones) in the whole body, which can be of extreme importance in the course of epilepsy. It has been found that, contrary to suppositions, the reduction in gut microbiota diversity due to KD does not lead to an intensified inflammatory condition. In a study of 2023, a normal level of S100A12 intestinal inflammation marker was demonstrated in children with DRE, who were on KD [144]. At the same time, one study demonstrated that the development of mild convulsions in children was preceded by a mild gastroenteritis [95], while inflammatory intestinal diseases themselves are also associated with seizures [106]. A study from 2022 demonstrated that in children with DRE, due to KD application, out of 96 inflammation markers, the values of as many as 26 were significantly decreased (among other markers, IL-17A, IL-17C, TNF, IL-12B, IL-18R1 and GDNF) and only those of three markers were increased (CCL25, IL-18 and IL-1 alpha), while the increase in IL-18 value was associated with a possible anti-inflammatory protection of the large bowel [147,172]. An anti-inflammatory effect on the intestine can be exerted by increased amounts of short-chain fatty acids (SCFA) in the intestine due to KD application, which has been confirmed in a study conducted for six months [149]. In the abovementioned studies, a strong relationship was demonstrated between the effects observed and the microbiota and specific genera of intestinal bacteria. Ketogenic diet can cause a reduction in inflammatory conditions in many ways. It has been demonstrated, among other findings, that butyrate (SCFA) acts synergistically with βOHB, reducing intestinal inflammatory conditions in children with inflammatory bowel disease (IBD) and inhibiting histone deacetylase (HDAC), reducing inflammatory cytokine production and increasing H3 histone acetylation in macrophages. In view of that, the synergy of butyrate and βOHB epigenetically reduces the inflammatory condition of the intestinal mucosa. KD in children with IBD can lead to an increase in the SCFA-producing bacteria (among other organisms, *Bifidobacterium* spp., *Lactobacillus* spp., *Bacteroides* spp., *F. prausnitzii* and R. intestinalis) [173]. It has also been shown that the microbiome modulation, specific for KD, decreases the level of intestinal proinflammatory Th17 cells [139]. Studies demonstrated that ketogenic diet was also related to a significantly greater reduction in inflammatory conditions caused by acute endotoxemia, compared with the control diet, after which a five times higher expression of hepatic NFκB and higher IL-6 and TNF-α concentrations were noted [174]. Moreover, it is known that lipopolysaccharide-induced endotoxemia causes a higher proneness to convulsions, increases BBB permeability and cerebral level of proinflammatory cytokines [175]. In the analysis of anti-inflammatory mechanisms of KD, inhibition of the NLRP3 signalling pathway is also an important aspect [176].

It has been hypothesized that microglia are closely associated with sterile neuroinflammation and that hyperactivation of the NLRP3 inflammasome/IL-1 axis in microglia generates a pro-inflammatory and pro-convulsant endo-environment, which contributes to the initiation of FIRES [177].

In a study of human focal epilepsy, investigators found an increased number of NLRP3-expressing CD3+ and CD14+ cells in peripheral blood mononuclear cells from epilepsy patients. Activation of NLRP3 inflammatory vesicles can increase the release of pro-inflammatory cytokines [178]. In a clinical trial in healthy subjects, it was found that 3-day short-term KD significantly reduced interleukin 1 beta (IL-1β) and TNF-α secretion induced by adenosine triphosphate or palmitate stimulation in human macrophages, and that beta-hydroxybutyric acid (BHB), the main product of KD, could exert anti-inflammatory effects by inhibiting the activation of the NLRP3 inflammatory vesicle and its associated signaling pathway [179]. In patients with epilepsy, the correlation of the set of micro-organisms specifically associated with the development, course, and treatment of the disease and response to ketogenic diet, is yet another mechanism. It is known that some, but not all, specific bacteria, as part of microbiome modulation by KD, are correlated with the simultaneously occurring antiepileptic effect. In their study of 2022, Dahlin et al. demonstrated a correlation between B. longum, B. breve and TNF-alpha. All levels were higher in children who later applied ketogenic diet and achieved results in the form of a reduction in seizure frequency, compared with children who did not respond to KD. The authors clearly conclude that they can be used as biomarkers for the identification of individuals potentially susceptible to the treatment. The antiepileptic response to KD in children with DRE was also associated with the presence of *Gordonibacter pamelaeae, Eggerthella lenta* and *Lactococcus lactis*, while *Eubacterium rectale* and *Alistipes shahii* were associated with a lack of response [147]. In the study, a positive correlation between several *E. coli* strains and both CCL25 and IL-18 and also a negative correlation between CCL25 and other *Bifidobacteria* (*B. angulatum, B. adolescentis, B. kashiwanohense*) and B. breve were found. In another publication, increased abundance of *Parabacteroides* and *Akkermansia municiphila* was associated with antiepileptic effect and increased hippocampal GABA/glutamate levels [87]. The mentioned bacteria exert so a strong effect that they reduce the frequency of seizures even in mice on a normal diet. An association was also demonstrated of *Bacteroides fragilis*, as the bacterium of key importance, with the antiepileptic effect of ketogenic diet, while *Enterococcus faecium, Bifidobacterium longum* and *Eggerthella lenta* were suggested as biomarkers of incurable epilepsy [143,180]. A number of differences in the microbiome after KD application in epileptic patients have caused that the authors in their publications to clearly conclude that specific gut microflora can serve as a biomarker of effectiveness and even as a potential therapeutic goal in patients with DRE [150].

**Figure 1:**
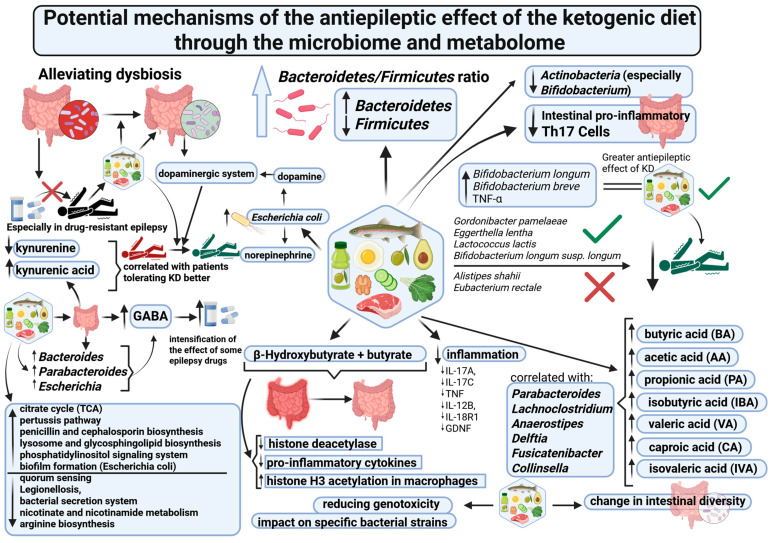
Potential mechanisms of the antiepileptic effect of the ketogenic diet through the microbiome and metabolome. Created in BioRender Dyńka D., Paziewska A. (2025). YT293XN14B.

Other mechanisms may include an effect of ketogenic diet on metabolic pathways at gut microbiome level [181]. In one study, an effect of KD was demonstrated at 3 levels of the Kyoto Encyclopaedia of Genes and Genomes (KEGG). Differences were found in 320 pathways, which could be affected by KD in epileptic patients, as described in chapter “5.1”. Among other pathways, that concerns the metabolic pathway of purines, which play an important role, among other functions, in signal transmission. It is also known that KD increases the levels of ATP and adenosine, which may be the main mediators of the neuroprotective effect of the diet [142,182,183]. An effect of KD was also demonstrated on the pathways related to the metabolism of carbohydrates [118]. Other mechanisms include the effect mediated by the bacterial metabolites (such as short-chain fatty acids and production of neurotransmitters), metabolism of tryptophan, intestinal hormonal signalling, neuroendocrine hypothalamic–pituitary–adrenal (HPA) axis, immune system (including intestinal one), intestinal/vagus nerves, relations between intestinal mucosal barrier and blood–brain barrier [171,184,185,186]. KD can also exert a significant effect in epilepsy through a reduction in fecal water genotoxicity, what has been demonstrated in one publication [148]. An integral part of ketogenic diet effect in epilepsy is its influence through the gut–brain barrier, which participates in a significant proportion of the described mechanisms of action [187]. Potential mechanisms of the antiepileptic effect of the ketogenic diet through the microbiome and metabolome are illustrated in Figure 1.

## 6. Strengths and Limitations

The article provides a comprehensive narrative review integrating nearly 200 studies describing the role of the microbiome in epilepsy, including both taxonomic and functional aspects, the impact of the ketogenic diet on the microbiome, and how ketogenic diet–induced changes in the microbiome may influence epilepsy, while also considering clinical studies in patients with epilepsy and analyzing mechanistic evidence linking the ketogenic diet, epilepsy, and the microbiome, making this review a valuable resource for researchers and clinicians.

This review has several limitations. First, the included studies are heterogeneous, with many having small sample sizes, varying ketogenic diet protocols, or lacking long-term follow-up, which limits the generalizability of the findings. Second, mechanistic evidence remains incomplete, as direct causal studies, such as fecal microbiota transplantation in humans—are still limited. Third, the role of microbial metabolites like TMAO in ketogenic diet–treated epilepsy is poorly understood, as are the long-term effects of the diet on gut health, including the potential risk of dysbiosis after diet discontinuation. Furthermore, it is important to note that this is a narrative review rather than a systematic one, and therefore it does not include all available evidence on every aspect. On the other hand, a narrative approach was the only feasible way to integrate such a large and diverse body of literature and to provide a comprehensive overview of this complex topic.

## 7. Conclusions

Summarising the available evidence related to microbiome and metabolome modulation through a ketogenic diet in patients with epilepsy, a conclusion can be surely drawn that it is a potential therapeutic mechanism in the disease. This results from the presence of altered gut microbiota in epileptic patients (particularly with DRE), which is correlated with the course of the disease and frequency of seizures, which, in turn, can be modulated by a ketogenic diet. The observed relationship between ketogenic diet application and microbiome and metabolome changes, with the simultaneous strong antiepileptic effect, strengthens the position of that mechanism as possibly belonging to the key ones in the treatment of epilepsy. Besides the significant correlations between microbiome and metabolome and the course of epilepsy in patients on KD, the observed results are correlated with a number of mechanisms. They concern, among other effects, an alleviation of dysbiosis, reduction in inflammatory conditions in the intestine (and the whole body), effect on the brain–gut barrier, mediation in the production of neurotransmitters (GABA, serotonin) and kynurenine, effect on the dopaminergic system and on many metabolic pathways at the microbiome level, production of SCFA, effects of specific bacterial strains, and inhibition of genotoxicity. It should be emphasized that identification of specific bacterial strains may be both a potential diagnostic marker or a predictive marker in the treatment of DRE. Moreover, it could also possibly help to develop new therapies (probiotics) for the modulation of microbiome and metabolome and thus for the reduction in signs of epilepsy even during infancy. However, the ketogenic diet in pediatric epilepsy has its limitations; therefore, to minimize them, it should be implemented under the supervision of a specialized physician and dietitian. This approach helps reduce the risk of potential nutrient deficiencies, adverse effects, and the challenges associated with maintaining long-term adherence to the diet.

In summary, based on published studies and the collected observations, it can be concluded that this is a highly promising potential mechanism of action of the ketogenic diet in the treatment of epilepsy. It may be a trendsetting and extremely fruitful direction of studies on the treatment of epilepsy; therefore, the need for further research in this field is justified, in particular, in both short- and long-term follow-up.

## Figures and Tables

**Table 1:** Characteristics of microbiome and metabolome in epileptic patients.

Year of Study, Reference	Study Aim	Groups	Results
2025 [116]	To investigate gut microbiota differences between medication-resistant and medication-sensitive pediatric epileptic patients with genetic and presumed genetic etiology.	1. 20 children with medication-resistant (MR) epilepsy 2. 21 children with medication-sensitive epilepsy 3. 27 age-matched healthy controls (HCs)	1. Significant differences between the ratio of *Bacteroidetes* and *Firmicutes* were not found. 2. Significant dominance in Hung in the MR population. 3. BD measures indicate that a unique gut bacterial profile was present in patients with epilepsy, but no significant differences were found between MR and MS subgroups.
2023 [122]	Elucidate the mechanistic roles of the gut microbiome in epileptogenesis following cerebral palsy and identify gut microbiota alterations related to seizure control	1. 8 children with non-epileptic cerebral palsy (NECP) 2. 13 children with cerebral palsy with epilepsy (CPE) (including 5 with drug-resistant epilepsy (DRE))	1. In the CPE group, lower counts of *Bacteroides fragilis* and *Dialister invisus* and higher counts of *Phascolarctobacterum faecium* and *Eubacterium limosum* were found 2. In the DRE group, an increased count of *Veilonella parvula* was observed. 3. In the CPE group, an increase was found in the concentrations of metabolites, i.e., kynurenic acid, L-vinic acid, D-saccharic acid, 2-oxindole, dopamine, 2-hydroxyphenylalanine and 3,4-dihydroxyphenyloglycol. 4. In the DRE group, an increase was observed in indole and homovanilic acid concentrations. 5. In the CPE group, an increased number of ethanol production pathways but a decreased number of pathways of serine, glutamate, quinolinic acid, glycerol degradation and of sulphate and nitrate reduction were found.
2023 [123]	To investigate the activity of the gut–brain axis in the pathogenesis of childhood epilepsy and to define biomarkers capable of assisting with determining new strategies in that context.	1. 20 children with epilepsy of “unknown aetiology” 2. 7 healthy controls in the same age group	1. Differences between the groups were observed at genus, order, class, family and phylum levels. 2. Only in the group of epileptic patients *Megamonas* and *Coriobacterium* were observed. 3. Only in the control group *Flavihumibacter, Niabella, Anoxybacillus, Brevundimonas, Devosia* and *Delftia* were found.
2022 [120]	To characterize the fecal microbiome, investigate the differences between epilepsy patients and healthy controls, and evaluate the potential efficacy of the fecal microbiome as a diagnostic tool for epilepsy.	1. 24 patients with epilepsy (EPs) 2. 50 healthy people in the control group (HCs)	1. In the group of EPs, a lower alpha diversity (mean Shannon Index value = 2.53 (epilepsy) vs. 3.4 (control group) was found. 2. In the EPs group, a significant increase was observed in *Proteobacteria* and *Actinobacteriota* (at phylum level) and 23 bacterial genera, i.e., among other organisms *Faecalibacterium, Escherichia-Shigella, Subdoligranulum* and *Enterobacteriaceae* (unclassified). 3. In the group of HCs, the relative abundance increased of *Bacteriota* (at phylum level) and 59 bacterial genera, i.e., among other bacteria *Bacteroides, Megamonas, Prevotella, Lachnospiraceae* (unclassified) and *Blautia.*
2022 [124]	Exploring the correlation between gut and oral microbiota in children with cerebral palsy and epilepsy (CPE).	27 children with cerebral palsy and epilepsy (CPE).	1. *Bifidobacterium*, *Bacteroides* and *Prevotella* were noted as the most frequent bacterial genera in children with CPE. 2. A close correlation was found between microbiota in the oral cavity and gut microbiota. An abnormal oral microbiota can lead to dysbiosis of the gut microbiota.
2021 [121]	A comparison of the gut microbiota among adult patients with drug-responsive and drug-resistant epilepsy	1. 23 patients with drug-sensitive epilepsy (DSE) 2. 21 patients with drug-resistant epilepsy (DRE)	1. No significant differences in alpha and beta diversity 2. In the DSE group, an increase in the relative abundance of *Bacteroides fragilis* and *Ruminococcus_g2* was found 3. In the DRE group, an increase in the relative abundance of *Negativicutes* (*Firmicutes* phylum) was seen
2020 [114]	To explore the structure and composition of the fecal microbiota of patients with epilepsy.	1. 55 patients with epilepsy (EPs) (including 30 with drug-resistant epilepsy (DRE)) 2. 46 healthy people in the control group (HCs)	1. The alpha diversity was significantly lower among EPs (the mean number of the found species = 275.33 ± 41.64 vs. 347.26 ± 102.40 in the HC group). 2. In the group of EPs, an increase in *Actinobacteria* and *Verrucomicrobia* and a decrease in *Proteobacteria* (at phylum level) occurred, while at genus level a decreased abundance was found of *Klebsiella, Sutterella, Escherichia-Shigella, Lachnospiraceae_NK4A136*_group and *Lachnoclostridium*, and an increase in *Prevotella_9, Blautia, Bifidobacterium, Akkermansia, Megamonas, Ruminococcaceae_UCG_014, Ruminococcus_gnavus*_group, *Romboutsia* and *Eubacterium_hallii*_group was demonstrated. 3. In the DRE group an increase in *Actinobacteria, Verrucomicrobia, Nitrospirae* and *Firimicutes,* but a reduction in the *Cyanobacteria* phylum were noted. At the genus level an increase occurred in *Blautia, Bifidobacterium, Subdoligranulum, Dialister* and *Anaerostipes*, while a reduction in *Parabacteroides* was found.
2019 [118]	Comparison of gut microbiome composition in epileptic children vs. the control group and assessment of ketogenic diet effect on the taxonomic and functional composition of microbiota in epileptic children	1. 12 children with drug-resistant epilepsy (DRE) 2. 11 healthy people in the control group (HCs)	1. In the DRE group, an evidently lower total number of the observed metagenomic operational taxonomic units (mOTU), of total Chao1 species richness and Shannon evenness index were noted. 2. In the DRE group, a lower alpha diversity and a higher beta diversity were observed, compared to the HCs.
2018 [117]	Exploring whether dysbiosis is involved in the mechanism of drug-resistant epilepsy.	1. 42 patients with drug-resistant epilepsy (DRE) 2. 49 patients with drug-sensitive epilepsy (DSE) 3. 65 healthy people in the control group (HCs)	1. In the DRE group, an increased alpha diversity was found (particularly in the subgroup of patients with four or more seizure attacks yearly). 2. In the DRE group, an increase was observed in the bacteria belonging mainly to the *Firmicutes* phylum, i.e., *Roseburia, Coprococcus, Ruminococcus* and *Coprobacillus*, compared to the DSE group. 3. In the DSE group, the gut microbiota composition was similar to that in the healthy control group.
2017 [119]	To investigate whether patients with refractory epilepsy and healthy infants differ in gut microbiota (GM), and how ketogenic diet (KD) alters GM.	1. 14 infants with drug-resistant epilepsy (DRE) 2. 30 healthy infants in the control group (HCs)	1. In the DRE group, the greatest shares were of the phyla *Firmicutes* (45.82%), *Bacteroidetes* (26.75%), *Proteobacteria* (24.34%), *Actinobacteria* (2.38%), *Verrucomicrobia* (0.59%) and *Fusobacteria* (0.09%). At the genus level, *Cronobacter predominated* (23.30%) (compared to 0% in HCs) 2. In the group of HCs, the greatest shares were of the phylum *Bacteroidetes* (53.01%), followed by *Firmicutes* (34.38%), *Actinobacteria* (8.49%), *Proteobacteria* (2,9%), *Verrucomicrobia* (0.78%) and *Fusobactera* (0.43%). At the genus level, *Bacteroides* predominated (42.68%) (compared to 17.93% in the DRE group), but also *Prevotella* (7.29% vs. 0.37%) and *Bifidobacterium* (7.84% vs. 0.91%) were increased 3. In the group of HCs, a higher diversity of the microbiota was noted (Shannon index analysis)

**Table 2:** Effect of ketogenic diet on the modulation of gut microbiome and metabolome in epileptic patients.

Year of Study, Reference	Study Aim	Groups	Results
2023 [143]	Exploring the relationship and potential altered pathways between ketosis, gut microbiota, and mitochondrial epilepsy.	1. Ketogenic diet group (KD)—8 patients with mitochondrial epilepsy 2. Control group (CD)—7 patients with mitochondrial epilepsy	1. Higher diversity of gut microbiota in the control group (significantly higher acc. to Chao1 diversity index, insignificantly higher acc. to Shannon diversity index) 2. In the KD group, a percentage reduction in *Firmicutes* (42.76% vs. 48.13% in CD) and an increase in *Bacteroidota* (36.93% vs. 25.41% in CD) were observed. 3. In the KD group, a percentage reduction in *Actinobacteriota* (1.66% vs. 7.64% in CD), *Fusobacteriota* (0.68% vs. 1.65% in CD) and *Desufobacterota* (0.15% vs. 0.50% in CD) was found. 4. In the KD group, a percentage increase was seen in *Bacteroides* (28.78% vs. 9.51% in CD) (mainly *Bacteroides fragilis*), *Blautia.s_Blautia_sp_N6H1_15* and *Anaerotignum_lactatifermentans* species 5. In the KD group at 3 KEGG level an increased enrichment was noted in pathways, i.e., citrate pathway (TCA), pertussis pathway, biosynthesis of penicillin and cephalosporins, biosynthesis of lysosomes and glycosphingolipids, phosphatidylinositol signalling system, biofilm formation (*Escherichia coli*) and a reduced enrichment in the KD group was observed in the pathways including Quorum sensing, Legionellosis, nicotinate and nicotinamide metabolism, arginine biosynthesis or Bacterial secretion system.
2023 [145]	Effects of the ketogenic diet on gastrointestinal function, gut microbiome, inflammation, and quality of life in children with intractable epilepsy	1. Ketogenic diet group (KD)—14 patients with intractable epilepsy (IE) 2. Control group (CD)—7 patients with intractable epilepsy (IE)	1. In the KD group a lower diversity of microbiota was noted 2. Both groups had normal levels of S100A12 (a marker of enteritis).
2022 [147]	To analyse changes in the fecal microbiota and levels of inflammation markers in blood after three months on KD treatment in a cohort of children with pharmaco-resistant epilepsy.	28 children with pharmaco-resistant epilepsy.	1. *Bifidobacterium longum* and *Bifidobacterium breve* were significantly correlated with TNF-alpha concentration. The levels of all three were increased in children, who later responded with a reduced frequency of seizures (compared to children not responding to KD). 2. *B. kashiwanohense PV20-2, B. angulatum GT102, B*. *adolescentis ATCC 15703* and *B. breve JCM 7019* demonstrated negative correlations with CCL25 3. The values of 26 inflammatory condition markers decreased in the consequence of KD application (among other markers, IL-17A, IL-17C, TNF, IL-12B, IL-18R1 and GDNF). 4. The values of three inflammatory condition markers increased in the consequence of KD application (CCL25, IL-18 and IL-1 alpha).
2021 [148]	To investigate whether 1 month of KD affects the gut environment in epileptic patients, by analysing short-chain fatty acids (SCFA) production and fecal water toxicity.	7 patients with drug-resistant epilepsy (DRE)	After ketogenic diet application a significant reduction was found in: -fecal water genotoxicity level (from the mean value 33.4 (32.0–41.8) to 29.2 (26.2–32.6)) (expressed as % of DNA in the tail) -total SCFA amount (from the mean value 20.7 mg/g to 9.3 mg/g) -acetoacetate amount (from the mean value 8.4 mg/g to 2.7 mg/g) -butyrate amount (from the mean value 4.8 mg/g to 3.2 mg/g) -propionate amount (from the mean value 3.4 mg/g to 2.4 mg/g) -isovalerate amount (from the mean value 1.1 mg/g to 0.6 mg/g) -isobutyrate amount (from the mean value 0.6 mg/g to 0.3 mg/g)
2021 [149]	To investigate the composition of the intestinal microbiota and its association with fecal short-chain fatty acids (SCFAs) in children with drug-refractory epilepsy (DRE) before and after treatment with a ketogenic diet (KD).	1. 12 children with drug-resistant epilepsy (DRE) 2. 12 people in the control group (CG)	After ketogenic diet application an increase was noted in: -SCFA amount in feces -the abundance of *Subdoligranulum, Dialister, Alloprevotella* and a decrease of: -the abundance of *Bifidobacterium, Akkermansia, Enterococcaceae* and *Actinomyces*
2019 [118]	Comparison of gut microbiome composition in epileptic children vs. the control group and assessment of ketogenic diet effect on the taxonomic and functional composition of microbiota in epileptic children	1. ketogenic group (KD)—12 children with drug-resistant epilepsy (DRE) 2. 11 healthy people in the control group (HCs)	In the KD group the following were observed: -reduction in alpha diversity (mOTU, Chao1 and Shannon indices) -reduction in relative abundance of *Actinobacteria* (mainly *Bifidobacterium*, particularly two species: *Bifidobacterium longum* (reduction from 8.1% to 2.4%) and B. adolescentis (reduction from 3.2% to 0.2%)) and increase in *Proteobacteria.* -a reduction in the abundance of *Eubacterium rectale* (from 2.5% to 0.5%) and *Dialister genus* (from 2.2% to 0.4%), and an increased abundance of *Escherichia* genus (from 3.1% to 8.5%) (mainly *Escherichia coli*). -changes in 29 SEED subsystems (among other changes, reduction in seven pathways engaged in the metabolism of carbohydrates)
2018 [150]	To investigate the characteristics and composition of intestinal microbiota in children with refractory epilepsy after ketogenic diet (KD) therapy and to explore the bacterial biomarkers related to clinical efficacy.	20 children with refractory epilepsy	KD application led to: -a reduction in alpha diversity of gut microbiota -an increase in the abundance of *Bacteroidetes* and a significant decrease in the abundance of *Firmicutes*. -in the group not responding to KD, an increase occurred in the abundance of *Clostridiales, Rikenellaceae, Lachnospiraceae, Ruminococcaceae*, and *Alistipes* (compared to individuals responding to the treatment).
2017 [119]	To investigate whether patients with refractory epilepsy and healthy infants differ in gut microbiota (GM), and how ketogenic diet (KD) alters GM.	1. 14 infants with drug-resistant epilepsy (DRE) 2. 30 healthy infants in the control group (HCs)	After a week on KD, at the phylum level, the following were found: -an increase in *Bacteroidetes* (38.71% vs. 26.75% before) and *Fusobacteria* levels (0.32% vs. 0.09% before). -a reduction in *Proteobacteria* level (10.77% vs. 24.31% before). After a week on KD, at the genus level, the following were found: -a decrease in the percentage of *Cronobacter* (10.44% vs. 23.30% before), *Erysipelatoclostridium* (4.89% vs. 8.67%), *Faecalibacterium* (4.41% vs. 8.59% before) and other organisms, including *Streptococcus, Alistipes, Veillonella, Bifidobacterium, Lachnoclostridium, Lactobacillus* -an increase in the percentage of *Bacteroides* (24.42% vs. 17.93% before), *Blautia* (7.69% vs. 2.57% before), *Gemmiger* (5.05% vs. 1.92% before), *Dysgonomonas* (5.36% vs. 1.49% before) and other bacteria, including *Anaerostipes, Prevotella, Dorea and Odoribacter*

## Data Availability

No new data were created or analyzed in this study. Data Sharing is not applicable to this article.

## Abbreviations

KD	ketogenic diet
SCFA	short-chain fatty acids
MAD	Modified Atkins Diet
DRE	drug-resistant epilepsy
MCTs	monocarboxylate transporters
BBB	blood–brain barrier
GABA	gamma-aminobutyric acid
KATP	ATP-sensitive potassium
BA	butyric acid
AA	acetic acid
PA	propionic acid
IBA	isobutyric acid
VA	valeric acid
CA	caproic acid
IVA	isovaleric acid
TMA	trimethylamine
GPC	glycerophosphorylcholine
TMAO	trimethylamine N-oxide
DSE	drug-sensitive epilepsy
mOTU	metagenomic operational taxonomic units
PCA	the principal component analysis
OTUs	operational taxonomic units
CD	control group
KEGG	Kyoto Encyclopaedia of Genes and Genomes
KYN	kynurenine
KYNA	kynurenic acid
3-OH-KYN	3-OH-kynurenine
IBD	inflammatory bowel disease
HDAC	inhibiting histone deacetylase
